# Quo vadis^1^ computational analysis of PPI data or why the future isn't here yet

**DOI:** 10.3389/fgene.2015.00289

**Published:** 2015-09-15

**Authors:** Konstantinos A. Theofilatos, Spiros Likothanassis, Seferina Mavroudi

**Affiliations:** ^1^InSyBio Ltd.London, UK; ^2^Pattern Recognition Laboratory, Department of Computer Engineering and Informatics, University of PatrasPatras, Greece; ^3^Department of Social Work, School of Sciences of Health and Care, Technological Educational Institute of Western GreecePatras, Greece

**Keywords:** protein–protein interactions, protein–protein interaction networks, protein clusters, protein functions, biomarker discovery, drug discovery

## Introduction

Proteins have been proven to be among the most significant cellular molecules as they participate in most cellular functionalities. Researchers have deployed a variety of experimental methods for the identification of Protein–Protein Interactions (PPIs). The emergence of high-throughput experimental techniques for the prediction of PPIs, revealed a wide range of PPIs in many organisms. This information alongside with information from small scale experimental techniques has been stored in public available databases and repositories. It is well-known that experimental data include many false positive predictions and provide only low coverage on the full interactomes. This fact has led to the design and development of many computational methods for the prediction of PPIs (Theofilatos et al., 2011).

The experimental PPI data have been extensively used in many studies during the last decades and their availability gave a significant boost in training new algorithmic models for the prediction of PPIs and in the overall analysis of PPI data.

Despite the promising results of algorithmic solutions for PPIs' analysis which fostered molecular biology research, in our opinion the research on computational methods for analyzing PPI data has been recently stagnated. Using the online tool MLtrends introduced by Palidwor and Andrade-Navarro (2010) in a preliminary investigation we have observed that the publications related to the search term “*protein–protein interactions” AND analysis* (abstract and title were searched for this term) present a constant increase in absolute numbers. However, when applying normalization by dividing with the total number of annual publications, we observe a relatively stable percentage of publications related PPI analysis in the last decade. In contrast, systems biology publications present a big positive slope in the last decade even when normalized by the total number of annual publications. This diversification shows that even if the actual total number of publications related to PPIs analysis is increasing as the total number of scientific journals is increasing in the last decade, their total impact on the systems biology domain is decreasing. Additionally, only a few PPI based research works have been published lately with significant impact in clinical research and translational bioinformatics.

In this paper, first we summarize the developments on computational analysis of PPI data and second, we present our belief about the future of PPI data analysis emphasizing in presenting the constraints that have delayed the transition from the current methodologies to a holistic bioinformatics approach, for linking biological and clinical data. Specific solutions are also proposed for all these constraints in order to achieve the optimal exploitation of PPI bioinformatics' approaches.

## State-of-the-art and recent advancements of the computational analysis of PPI data

A wide range of computational methodologies and tools have been proposed during the last decade for the analysis of PPI data. These methods are emphasizing on algorithmic solutions for the problems of predicting and scoring PPIs, the construction of PPI networks, the prediction of protein complexes, and the functional annotation of proteins. The results of these solutions have been uploaded to public available databases and many tools have been supplied to the molecular research scientific community enabling the analysis of the PPIs from a single organism in a few days. State of the art computational methods for the prediction of PPI combine information from different sources and have presented adequate classification performance. Recent approaches (Zhang et al., 2012; Saha et al., 2014; Theofilatos et al., 2014) have attempted to overcome the bottlenecks in this PPIs prediction, namely the definition of negative datasets, the feature selection, the class imbalance, the tradeoff between classification performance and interpretability, missing features values and the calculation of a confidence score for every PPI. The advancements on the computational prediction and scoring of PPI enabled the construction of binary PPI networks with increased coverage on the full interactome. Many tools have been developed so far offering efficient and interactive visualization of large PPI networks (Smoot et al., 2011; Li et al., 2013; Tripathi et al., 2014). As a next step, significant results have been extracted from the analysis of PPI graphs. This analysis includes methods for predicting protein complexes (Chen et al., 2014) and the functional and topological characterization of proteins (Ma et al., 2014). Recent approaches (Nepusz et al., 2012; Hanna and Zaki, 2014; Theofilatos et al., 2015) have attempted to face with some of the remaining challenges for the prediction of protein complexes such as the prediction of overlapping clusters and the management of weighted PPI graphs thus increasing the accuracy having limited prior knowledge for known protein complexes. One of the ultimate goals of PPI data analysis is to functionally characterize proteins and their interactions. The main limitation of protein function prediction until now is that a combined framework to characterize the full proteome functionally of a single organism having a meaningful confidence score for every annotation does not exist. However, with the continued development of new algorithms and the improvement of experimental techniques we strongly believe that this will be achieved in the next few years.

## Bottlenecks in computational analysis of PPI data and reasons for its reduced impact in pharmacy and medicine

Computational methodologies for the analysis of PPIs undoubtedly contributed to the advancement of systems biology research. In our opinion, however we have reached the point where research on novel computational methods has stagnated and further advancements in systems biology research cannot be achieved solely through the development of more sophisticated algorithmic solutions. Recently, many researchers have suggested that advancements in the field of PPIs research will be facilitated by improved integration of clinical and molecular data, introducing new clinical phenotype data, such as the ones coming from integrated data using the technologies of smart sensors and personalized medicine, in a format manageable to computational approaches (Tiffin et al., 2009). The recent advances in other fields of molecular biology, such as the next generation sequencing data and their analysis, has enabled the transformation of traditional bioinformatics to translation bioinformatics. Despite the large availability of works describing specific combinations of datasets to develop tools suitable for disease genes prioritization, “our understanding of how to perform useful predictions using multiple data sources or across biological networks is still rudimentary” (Nabhan and Sarkar, 2014), and in particular, to our knowledge, only a few systematic studies focused on the exploitation of integrated network methods in medicine applications (Schaefer et al., 2012; Vinayagam et al., 2014).

For all these reasons, a new approach and more ambitious objectives should be set for the analysis of PPI data in order to overcome all these limitations, to meet clinical needs and cover the lost space in translation bioinformatics analysis which has been gained by genomics and transcriptomics analysis. The main challenges for PPI analysis according to our opinion, except from the already mentioned data integration task and the linkage of PPI data with clinical data, are the incorporation of environmental information in the PPI data analysis, the extended study of PPIs among different organisms, e.g., host–pathogen interactions, the three dimensional reconstruction of 2D PPI networks for better representation of protein structure, isoforms and spatial information, the design of new methods for biomarker discovery using PPI data and the development of new methods which will facilitate drug discovery using PPI data. In addition, as firstly proposed by Lopes et al. (2011) and adopted by Schaefer et al. (2012) and Furlong (2013), the availability of condition-specific interactomes that are more representative of the interactions of the proteins in a given tissue or under certain conditions will improve the significance of such analysis. This could be done by providing more realistic results, especially for the exploration of human diseases, where the network topology properties of proteins encoded by disease genes in interactomes should be reassessed with spatiotemporal resolution in healthy and disease states.

Traditional PPI analysis' approaches study physical and functional PPIs without taking into account environmental influences which may strongly affect a PPI or even the formation of a protein complex. Specifically, it is known that the post translational modifications of proteins, which play an important role in enabling them to interact with other proteins, are significantly affected by environmental changes. Moreover, most complex diseases are attributed to generalized disturbances in genetic and proteomic level in cooperation with environmental causes. In order to exploit PPI analysis in medicine application, it should be combined with environmental information and one way to achieve this is the integration of metabolomics data.

Another field of PPI analysis, which has not yet been thoroughly explored, is the study of interactions between proteins from various organisms with a striking example being the interactions of proteins from host and pathogen organisms which play a significant role in the viability of the affected cells. Until recently there existed a lack of large scale efforts to analyze host–pathogen interactions (Krishnadev and Srinivasan, 2008). However, these data are now available and a few methods, such as (Kleftogiannis et al., 2015), for their analysis have already been published presenting the potential of this field.

One of the most significant new ideas is the one proposed by Garbutt et al. (2014). This idea refers to the prevalent two dimensional format of the PPI graphs which is oversimplified and may lead to loss of information. To take advantage of the dynamic nature of PPI data, a new three dimensional representation should be stated integrating protein structure, conformation, isoforms and spatial information. Several recent research works take advantage of this idea to incorporate atomic-level protein structure information in PPI networks (Das et al., 2014) in order to examine the structural principles of disease mutations over a PPI network, or even to elucidate the genetic and molecular mechanisms of underlying human diseases (Wang et al., 2012).

One of the ultimate goals of PPI analysis should be the biomarkers' discovery. PPI networks contain significant information for the cellular mechanisms and functionalities which should be exploited to uncover disturbances in a network level. The traditional methods which attempt to uncover biomarkers from genetic variations or differences in the expression level have limited applicability as they export a large number of biomarkers without being able to locate the cause of the disturbance. When studying diseases in a network level, the variations are smaller and network based biomarkers are most likely to represent the cause of the disease. For this reason, more emphasis should be given to methods for comparing networks and locating biomarkers from the disturbed proteins, protein interactions, and protein complexes. Preliminary reports on methods for biomarkers' discovery through PPI networks comparison, revealed a new controversial issue (Wang et al., 2011). There are some arguing that hubs in PPI networks are most likely to be found as biomarkers and others arguing against. This issue should be further studied and clarified in order to uncover the network metrics which are adequate to be used for biomarker discovery using PPI graphs.

Another field of PPI analysis which should be further reinforced is drug discovery through PPI data. PPI data analysis has a variety of applications in drug discovery so far (Engin et al., 2014). An interesting idea to reduce the possible complications of a potential drug is to target proteins which are interacting with the target protein but have reduced significance in the overall network topology or even are leafs of the PPI graph. Even more such ideas are required to be implemented in a novel way to exploit PPI networks and their topological characteristics in the drug design process.

In the last 5 years significant initiatives, such as ELIXIR-Data for Life (Crosswell and Thornton, 2012) and Global Alliance for Data Sharing (Hayden, 2013), have attempted to promote biological data sharing, provide the adequate infrastructures and bring together molecular biologists, bioinformaticians and clinicians in order to translate life science research mainly to medicine and bioindustries. These initiatives should be even more re-enforced and promoted in order to integrate productively the knowledge and experience of so different fields toward the realization of personalized medicine. These efforts will be eased by the expected universal adoption of electronic medical records standardization and omics translation to clinical medicine (Issa et al., 2014). However, the full clinical potential of these initiatives will still remain unexplored until they are formed in a network perspective that place them within the systems medicine context. Protein–protein interaction analysis will by nature play a significant role in this network-perspective formation.

## Conclusions

In this opinion article we have presented our belief about the future of PPI data analysis emphasizing in presenting the constraints that delayed the transition from the current methodologies to a holistic bioinformatics approach, for linking biological and clinical data. The main constraints that should be surpassed are the incorporation of environmental information, the host–pathogen PPI data analysis and the expansion of the traditional 2D representation of PPI networks with a more flexible and informative 3D one. These constraints are of equal importance and most of them should be surpassed in order to ease the exploitation of PPI analysis in clinical applications. Moreover, we have stated the most significant areas of clinical applications of PPI data analysis which are biomarkers and drug discovery, and we have proposed certain ideas for advancing PPI analysis in these fields. The next few years, a new boost of clinical data is expected through the new electronic health records and data coming from the developing technologies of smart sensors and personalized medicine (Groves et al., 2013; Yang et al., 2015) and the computational analysis of PPI data should be ready to exploit this boost.

### Conflict of interest statement

The authors declare that the research was conducted in the absence of any commercial or financial relationships that could be construed as a potential conflict of interest.

## References

[B1] Chen B. Fan W. Liu J. Wu F. X. (2014). Identifying protein complexes and functional modules—from static PPI networks to dynamic PPI networks. Brief. Bioinformatics 15, 177–194. 10.1093/bib/bbt039 23780996

[B2] Crosswell L. C. Thornton J. M. (2012). ELIXIR: a distributed infrastructure for European biological data. Trends Biotechnol. 30, 241–242. 10.1016/j.tibtech.2012.02.002 22417641

[B3] Das J. Fragoza R. Lee H. R. Cordero N. A. Guo Y. Meyer M. J.. (2014). Exploring mechanisms of human disease through structurally resolved protein interactome networks. Mol. Biosyst. 10, 9–17. 10.1039/C3MB70225A 24096645 PMC4061614

[B4] Engin H. B. Gursoy A. Nussinov R. Keskin O. (2014). Network-based strategies can help mono-and poly-pharmacology drug discovery: a systems biology view. Curr. Pharm. Des. 20, 1201–1207. 10.2174/13816128113199990066 23713773

[B5] Furlong L. I. (2013). Human diseases through the lens of network biology. Trends Genetics 29, 150–159. 10.1016/j.tig.2012.11.004 23219555

[B6] Garbutt C. C. Bangalore P. V. Kannar P. Mukhtar M. S. (2014). Getting to the edge: protein dynamical networks as a new frontier in plant–microbe interactions. Front. Plant Sci. 5:312. 10.3389/fpls.2014.00312 25071795 PMC4074768

[B7] Groves P. Kayyali B. Knott D. Van Kuiken S. (2013). The ‘Bigdata’ Revolution in Healthcare. McKinsey Quarterly.

[B8] Hanna E. M. Zaki N. (2014). Detecting protein complexes in protein interaction networks using a ranking algorithm with a refined merging procedure. BMC Bioinformatics 15:204. 10.1186/1471-2105-15-204 24944073 PMC4230023

[B9] Hayden E. C. (2013). Geneticists push for global data-sharing. Nature 498, 16–17. 10.1038/498017a 23739403

[B10] Issa N. T. Byers S. W. Dakshanamurthy S. (2014). Big data: the next frontier for innovation in therapeutics and healthcare. Expert Rev. Clin. Pharmacol. 7, 293–298. 10.1586/17512433.2014.905201 24702684 PMC4448933

[B11] Kleftogiannis D. Wong L. Archer J. A. Kalnis P. (2015). Hi-Jack: a novel computational framework for pathway-based inference of host-pathogen interactions. Bioinformatics 31, 2332–2339. 10.1093/bioinformatics/btv138 25758402

[B12] Krishnadev O. Srinivasan N. (2008). A data integration approach to predict host-pathogen protein-protein interactions: application to recognize protein interactions between human and a malarial parasite. In Silico Biol. 8, 235–250. 19032159

[B13] Li W. Kinch L. N. Grishin N. V. (2013). Pclust: protein network visualization highlighting experimental data. Bioinformatics 29, 2647–2648. 10.1093/bioinformatics/btt451 23918248 PMC3789550

[B14] Lopes T. J. Schaefer M. Shoemaker J. Matsuoka Y. Neumann G. Andrade-Navarro M. A.. (2011). Tissue-specific subnetworks and characteristics of publicly available human protein interaction databases. Bioinformatics 27, 2414–2421. 10.1093/bioinformatics/btr414 21798963

[B15] Ma X. Chen T. Sun F. (2014). Integrative approaches for predicting protein function and prioritizing genes for complex phenotypes using protein interaction networks. Brief. Bioinformatics 15, 685–698. 10.1093/bib/bbt041 23788799 PMC4271058

[B16] Nabhan A. R. Sarkar I. N. (2014). Structural network analysis of biological networks for assessment of potential disease model organisms. J. Biomed. Inform. 47, 178–191. 10.1016/j.jbi.2013.10.011 24211613

[B17] Nepusz T. Yu H. Paccanaro A. (2012). Detecting overlapping protein complexes in protein-protein interaction networks. Nat. Methods 9, 471–472. 10.1038/nmeth.1938 22426491 PMC3543700

[B18] Palidwor G. A. Andrade-Navarro M. A. (2010). MLTrends: graphing MEDLINE term usage over time. J. Biomed. Discov. Collab. 5, 1–6. 10.5210/disco.v5i0.2680 20333611 PMC2990277

[B19] Saha I. Zubek J. Klingström T. Forsberg S. Wikander J. Kierczak M.. (2014). Ensemble learning prediction of protein–protein interactions using proteins functional annotations. Mol. Biosyst. 10, 820–830. 10.1039/c3mb70486f 24469380

[B20] Schaefer M. H. Fontaine J. F. Vinayagam A. Porras P. Wanker E. E. Andrade-Navarro M. A. (2012). HIPPIE: integrating protein interaction networks with experiment based quality scores. PLoS ONE 7:e31826. 10.1371/journal.pone.0031826 22348130 PMC3279424

[B21] Smoot M. E. Ono K. Ruscheinski J. Wang P. L. Ideker T. (2011). Cytoscape 2.8: new features for data integration and network visualization. Bioinformatics 27, 431–432. 10.1093/bioinformatics/btq675 21149340 PMC3031041

[B22] Theofilatos K. Dimitrakopoulos C. Likothanassis S. Kleftogiannis D. Moschopoulos C. Alexakos C. (2014). The Human Interactome Knowledge Base (HINT-KB): an integrative human protein interaction database enriched with predicted protein–protein interaction scores using a novel hybrid technique. Artif. Intell. Rev. 42, 427–443. 10.1007/s10462-013-9409-8

[B23] Theofilatos K. Dimitrakopoulos C. Tsakalidis A. Likothanassis S. Papadimitriou S. Mavroudi S. (2011). Computational approaches for the prediction of protein-protein interactions: a survey. Curr. Bioinform. 6, 398–414. 10.2174/157489311798072981

[B24] Theofilatos K. Pavlopoulou N. Papasavvas C. Likothanassis S. Dimitrakopoulos C. Georgopoulos E.. (2015). Predicting protein complexes from weighted protein–protein interaction graphs with a novel unsupervised methodology: evolutionary enhanced Markov clustering. Artif. Intell. Med. 63, 181–189. 10.1016/j.artmed.2014.12.012 25765008

[B25] Tiffin N. Andrade-Navarro M. A. Perez-Iratxeta C. (2009). Linking genes to diseases: it's all in the data. Genome Med. 1:77. 10.1186/gm77 19678910 PMC2768963

[B26] Tripathi S. Dehmer M. Emmert-Streib F. (2014). NetBioV: an R package for visualizing large network data in biology and medicine. Bioinformatics 30, 2834–2836. 10.1093/bioinformatics/btu384 24928209

[B27] Vinayagam A. Zirin J. Roesel C. Hu Y. Yilmazel B. Samsonova A. A.. (2014). Integrating protein-protein interaction networks with phenotypes reveals signs of interactions. Nat. Methods 11, 94–99. 10.1038/nmeth.2733 24240319 PMC3877743

[B28] Wang X. Gulbahce N. Yu H. (2011). Network-based methods for human disease gene prediction. Brief. Funct. Genomics 10, 280–293. 10.1093/bfgp/elr024 21764832

[B29] Wang X. Wei X. Thijssen B. Das J. Lipkin S. M. Yu H. (2012). Three-dimensional reconstruction of protein networks provides insight into human genetic disease. Nat. Biotechnol. 30, 159–164. 10.1038/nbt.2106 22252508 PMC3708476

[B30] Yang J. J. Li J. Mulder J. Wang Y. Chen S. Wu H. (2015). Emerging information technologies for enhanced healthcare. Comp. Industry 69, 3–11. 10.1016/j.compind.2015.01.012

[B31] Zhang Q. C. Petrey D. Garzón J. I. Deng L. Honig B. (2012). PrePPI: a structure-informed database of protein–protein interactions. Nucleic Acids Res. 41, D828–D833. 10.1093/nar/gks1231 23193263 PMC3531098

